# Precision oncology in the age of AI: lessons from AI-driven drug discovery and clinical translation

**DOI:** 10.1038/s44276-026-00221-1

**Published:** 2026-04-15

**Authors:** Wonbeak Yoo

**Affiliations:** Genomic Medicine Research Center, Korea Research Institute of Bioscience and Biotechnology, Daejeon, Republic of Korea

## Abstract

Drug discovery has been constrained by extended timelines and high costs, as the cumulative requirements of preclinical validation, multi-phase clinical trials, and regulatory approval have been imposed. Recently, computational modeling has been explored as a supportive approach to accelerate the identification and refinement of therapeutic candidates. Proof-of-concept was provided in a phase 2a trial of a de novo–designed TNIK inhibitor in idiopathic pulmonary fibrosis, in which safety, tolerability, and pharmacodynamic target engagement were demonstrated, with a trend toward reduced functional decline. This study showed that AI-derived molecules can advance into human testing, but broader validation, mechanistic understanding, and regulatory alignment remain essential. In oncology, where tumor heterogeneity, clonal evolution, and therapeutic resistance continue to constrain durable clinical benefit, there is an increasing need for adaptive and data-informed drug discovery strategies. This Perspective reviews recent progress and limitations in AI-driven drug discovery and early clinical translation. It emphasizes how the clinical evaluation of an AI-generated TNIK inhibitor serves as an early translational reference and outlines practical strategies for integrating multi-omics data, federated model validation, and adaptive trial design to advance precision oncology–oriented therapeutics.

## Introduction to governance challenges in AI-driven drug discovery for oncology

Artificial intelligence (AI) has increasingly been positioned as a methodological approach applied in pharmaceutical research and development, including oncology, transitioning from a supportive analytical tool to a computational strategy used to assist therapeutic discovery. Traditionally, the development of new pharmacologic agents has been characterized by extended timelines—often exceeding a decade—and substantial financial investment, frequently surpassing USD 1 billion per approved therapy. In oncology, these requirements are further shaped by tumor heterogeneity, therapy resistance, and narrow therapeutic windows, reflecting the cumulative demands of preclinical experimentation, sequential phase-based clinical trials, and regulatory scrutiny, all of which have been associated with high attrition rates and escalating costs [1, 2].

A recent phase 2a randomized, placebo-controlled clinical trial published in Nature Medicine has marked a significant inflection point in the field of translational medicine [3]. In this study, a TNIK inhibitor, generated through de novo generative modeling, was evaluated in patients with idiopathic pulmonary fibrosis (IPF). Although conducted in a non-oncologic disease setting, TNIK is a signaling kinase implicated in Wnt/β-catenin–related pathways that are also relevant to tumor progression, making the study methodologically relevant to oncology drug discovery. This trial represents an early instance in which a small molecule identified using AI-supported compound generation advanced to clinical evaluation beyond preclinical proof-of-concept.

In the trial, adult participants with confirmed IPF were randomized to receive either the AI-generated TNIK inhibitor or placebo over a defined treatment interval. The primary endpoints were safety and tolerability, while exploratory endpoints included changes in forced vital capacity (FVC) and levels of fibrosis-associated biomarkers. The investigational agent was found to be well tolerated, with no dose-limiting toxicities observed. Pharmacodynamic analyses confirmed evidence of target engagement via modulation of TNIK-associated pathways in peripheral blood. Additionally, trends consistent with attenuation of pulmonary function decline were reported. Although the study was not powered to establish definitive clinical efficacy, demonstrated that compounds identified through AI-assisted approaches can achieve acceptable safety profiles and measurable biological activity in humans, benchmarks that remain difficult to attain in oncology drug development.

For clarity, several terms central to AI-enabled drug discovery are briefly introduced here. AlphaFold refers to a deep-learning system capable of predicting three-dimensional protein structures and their complexes with atomic-level accuracy, supporting structure-informed drug design, including for cancer-associated targets. Self-supervised learning describes algorithms that learn intrinsic data representations without manual labeling, allowing the use of large unannotated chemical and cancer-relevant multi-omics datasets. Federated learning denotes a distributed training approach in which models are developed collaboratively across institutions without sharing raw patient data, a framework of increasing relevance for multi-center oncology research, preserving privacy while improving generalizability.

**Fig. 1:**
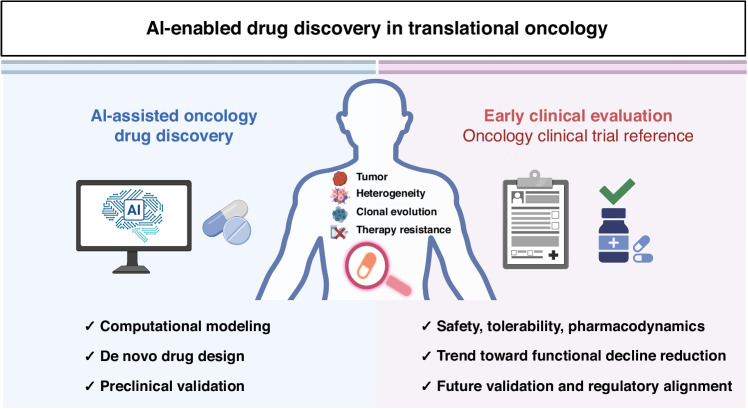
The left panel details the AI-assisted discovery process, utilizing computational modeling, de novo compound design, and preclinical validation to support early-stage therapeutic development. The central panel outlines critical biological challenges, including tumor heterogeneity, clonal evolution, and therapy resistance, which necessitate adaptive, data-driven discovery strategies. The right panel illustrates early-phase clinical evaluation as a translational reference point, summarizing safety, tolerability, and pharmacodynamic assessments that inform subsequent clinical validation and regulatory alignment. Together, this figure demonstrates the integration of computational approaches with experimental and clinical workflows to advance precision oncology. (Created with BioRender.com)

While these developments suggest methodological feasibility, a critical gap persists between algorithmic discovery and real-world translation. Previous reviews have largely focused on technical progress, while the clinical validation, regulatory evaluation, and governance frameworks required for integration into precision oncology remain less defined. This Perspective addresses that gap, with a specific focus on oncology, linking current examples with strategies to strengthen validation, transparency, and patient relevance (Fig. 1).

## Representative AI enabled therapeutics in clinical development

The TNIK inhibitor has joined a limited but expanding cohort of therapeutics developed through artificial intelligence-enabled platforms and advanced into clinical evaluation, reflecting a progressive transition from algorithmic discovery to patient-centered intervention. Several additional compounds exemplify this shift:

1. INS018_055 (Insilico Medicine): This anti-fibrotic agent, generated through a generative AI platform, has entered phase 2 clinical trials for idiopathic pulmonary fibrosis (IPF). It is one of the earliest AI-originated small molecules to progress beyond initial human safety assessment [4].
2. EXS21546 (Exscientia): A selective A2a receptor antagonist developed for immuno-oncology applications, this compound has completed phase 1 clinical evaluation and has been positioned for biomarker-guided efficacy trials [5]. This molecule was specifically optimized via AI to overcome adenosine-mediated immune suppression within the tumor microenvironment (TME), a critical barrier to achieving durable responses in solid tumors [6].
3. Baricitinib (BenevolentAI): Initially approved as a Janus kinase (JAK) inhibitor for rheumatoid arthritis, this agent was repurposed for COVID-19 treatment through AI-driven knowledge graph analysis. Its efficacy was subsequently confirmed in randomized clinical trials, leading to emergency use authorization by the U.S. Food and Drug Administration [7].

These examples reflect the expanding range of AI applications across the pharmaceutical landscape. They encompass de novo molecular design, indication expansion, and drug repurposing, applied across various therapeutic domains, including oncology, infectious diseases, and fibrotic disorders. From a developmental standpoint, these AI-enabled approaches have demonstrated the following translational advantages:

1. Acceleration of early-stage timelines: By automating key steps such as target identification, lead optimization, and in silico screening, these methods have enabled the compression of preclinical timelines from multiple years to several months [8].
2. Reduction in candidate attrition: Off-target effects, metabolic instability, and potential toxicities can be predicted computationally prior to compound synthesis, allowing high-risk candidates to be excluded at an early stage [9].
3. Enhancement of late-phase development: AI has been utilized to improve target validation and patient stratification, thereby increasing the likelihood of clinical success and regulatory approval [10].

Collectively, these developments indicate a broader methodological transition in the pharmaceutical sciences—from empirical iteration to computational rationalization. As these technologies continue to mature, they are expected to reshape the architecture of drug development pipelines, introducing greater efficiency, precision, and adaptability across a range of therapeutic indications.

## Technological drivers of AI-to-clinic translation

The maturation of AI-enabled drug discovery has been propelled by three interlocking technological advances that collectively shorten the path from in silico prediction to patient-ready therapeutics. First, advances in structure prediction—epitomized by AlphaFold 3—have extended high-accuracy modeling beyond static protein structures to encompass protein–ligand–RNA complexes at near-atomic resolution [9, 11]. This advancement has provided a more reliable foundation for structure-based drug design, enabling early-stage prioritization of candidates exhibiting favorable binding conformations prior to chemical synthesis. Second, self-supervised learning (SSL) has emerged as a key methodological innovation. Unlike traditional supervised frameworks, SSL enables the extraction of high-dimensional representations from vast and largely unlabeled chemical and multi-omics datasets. By doing so, it facilitates both target identification and lead compound generation, including in rare disease contexts where labeled data are limited or unavailable [12, 13]. Refocused the content on oncology by adding SSL-based examples using TCGA and ICGC datasets, along with relevant supporting references [14, 15]. Third, federated learning has allowed for the development of predictive models across multiple institutions without requiring the transfer of sensitive patient data. This decentralized learning architecture preserves local data governance while allowing model training on heterogeneous clinical datasets. As a result, the models benefit from improved generalizability, capturing a broader range of demographic and disease-specific variations reflective of real-world populations [16–18]. Collectively, these advances have redefined the methodological boundaries of pharmaceutical development. Artificial intelligence is no longer confined to preclinical modeling but is now positioned as an integrative platform capable of supporting regulatory-grade therapeutic development.

## Potential to reduce or supersede early stage experimental burden

A particularly transformative opportunity in drug development has been presented by the capacity to reduce— and in select cases, partially replace—early-stage in vitro and in vivo experimentation. Recent progress in high-throughput computational screening, multi-objective molecular optimization, and in silico absorption–distribution–metabolism–excretion–toxicity (ADMET) modeling has enabled the prioritization of compounds with favorable pharmacokinetic and pharmacodynamic profiles prior to synthesis [19–21]. Concurrently, computational models conceptually analogous to patient-specific “digital twins” have been under development to simulate individualized biological responses. These frameworks have allowed for the iterative refinement of therapeutic hypotheses in a purely computational setting [22, 23]. When effectively implemented, such approaches offer potential to lower preclinical attrition, reduce dependence on animal testing, and address longstanding ethical considerations in early-phase development.

Nevertheless, the reliability of these systems remains conditional upon empirical verification. Computational predictions remain vulnerable to algorithmic bias, overfitting, and the generation of spurious correlations. In this context, the most methodologically sound translational strategies have been characterized by a hybrid validation pipeline, in which candidates prioritized through in silico methods are subjected to expedited experimental confirmation, followed by recalibration of predictive models and targeted *in vivo* testing. This bidirectional framework is designed to safeguard translational rigor while preserving efficiency gains associated with computational acceleration [24–26].

## Challenges and risks in clinical translation

The TNIK inhibitor trial has demonstrated the initial feasibility of computationally guided therapeutic discovery; however, the transition from algorithm-based design to reproducible clinical benefit remains encumbered by several critical barriers.

### 1. Generalizability

The robustness of computational models must be established across demographically and clinically heterogeneous populations. Variability in diagnostic instrumentation, laboratory methodologies, and standard-of-care treatment algorithms has been shown to affect model performance. Without prospective, multi-center validation, candidates optimized within development datasets may fail to yield consistent efficacy or safety outcomes across broader clinical environments [27, 28].

### 2. Interpretability

The acceptance of AI-derived therapeutics within clinical and regulatory frameworks is contingent upon the provision of biologically plausible and mechanistically justified decision processes. Regulatory review necessitates traceable algorithmic logic, and clinical adoption further requires that therapeutic rationale be comprehensible to providers and patients alike—particularly in contexts involving high-risk interventions. Accordingly, interpretability has been regarded as a prerequisite for both safety oversight and stakeholder trust [29–31].

### 3. Equity and inclusivity

The underrepresentation of minority populations in training datasets has been associated with the propagation of therapeutic disparities. To ensure equitable benefit, foundational datasets must include diverse ethnic, socioeconomic, and geographic groups. Moreover, the integration of bias detection frameworks and population-specific genomic and environmental variables into model development is required to mitigate structural inequities in drug efficacy and tolerability [32–34]. This imperative is further amplified in oncology, where minority populations often face a disproportionate cancer burden, as they remain significantly underrepresented in the genomic datasets used to train AI models. Such data gaps risk overlooking population-specific variations in drug-metabolizing enzymes, such as cytochrome P450 (CYP450) isoforms, or distinct tumor mutational burdens that dictate therapy response [35, 36].

### 4. Regulatory harmonization

Current regulatory ecosystems exhibit substantial heterogeneity in their criteria for model validation, reproducibility assessment, and data interoperability. This fragmentation has introduced inefficiencies in international development and deployment of AI-enabled therapies. The establishment of harmonized global standards—for computational validation metrics, data sharing practices, and post-approval monitoring—has been identified as a critical step toward facilitating safe and timely market access [37–39]

In the absence of targeted mitigation strategies, these limitations may result in therapeutic candidates that meet technical performance metrics yet fall short of delivering sustained clinical impact. Addressing these challenges will be essential to realizing the translational potential of computational discovery methodologies and ensuring that algorithmically derived innovations contribute meaningfully to patient care [29, 40].

## Expanding AI’s role in precision oncology

The clinical evaluation of the TNIK inhibitor illustrates the feasibility of AI-generated therapeutics. From an oncology perspective, the next step is to assess how such approaches can be applied to individualized cancer care, where molecular heterogeneity and adaptive resistance complicate treatment selection. Integrating genomic, transcriptomic, proteomic, metabolomic, and spatial data with structural modeling can refine disease classification and guide oncology-focused therapeutic design [41–46]. Combined with federated validation and adaptive trial models, such integration supports scalable precision medicine. These approaches are especially relevant in oncology, where tumor heterogeneity and treatment resistance continue to limit durable outcomes [47, 48].

Moreover, the implementation of federated validation strategies has allowed for distributed model training and performance assessment across institutional boundaries without the need for centralized data aggregation. Through this decentralized paradigm, advanced modeling capabilities have become accessible to institutions with varying levels of computational infrastructure, while preserving patient privacy and regulatory compliance [49, 50]. In the context of oncology, these approaches are especially relevant, as tumor heterogeneity, clonal evolution, and treatment resistance continue to limit durable responses [45, 51, 52].

AI-enabled platforms that integrate spatial transcriptomics, immunogenomics, and longitudinal clinical data from cancer patients may support refined patient stratification, facilitate the prediction of resistance pathways, and guide the design of adaptive therapeutic regimens [53–57]. Furthermore, the integration of AI with liquid biopsy data—including circulating tumor DNA (ctDNA)—enables the real-time monitoring of minimal residual disease (MRD) and the early detection of resistance mutations, offering a window for AI-optimized adaptive treatment switching [58, 59]. Such applications highlight the methodological relevance of computational discovery frameworks for addressing the biological complexity of cancer, and for informing precision oncology at both the individual and population levels [60, 61].

Collectively, the integration of large-scale molecular profiling with real-time clinical data streams in oncology research and care has laid the groundwork for iterative precision therapeutic development [62, 63]. Within this adaptive framework, candidate compounds may be iteratively refined in response to evolving biomarker profiles, clinical outcomes, or resistance signatures. As a result, the conventional linear model of anticancer drug development is increasingly complemented by feedback-informed strategies that maintain alignment between therapeutic design and emergent patient data—thereby enhancing clinical relevance and translational efficiency.

## Conclusion: from proof of concept to clinical impact toward future oncology

The phase 2a evaluation of a de novo–designed TNIK inhibitor has been regarded as an early clinical reference point in the trajectory of computationally enabled drug discovery, illustrating the feasibility of translating in silico–derived therapeutic candidates into human studies. Within this trial, essential pharmacological benchmarks—including safety, pharmacodynamic target engagement, and early signals of efficacy—were achieved, supporting further clinical investigation. However, this milestone should be interpreted as an initial demonstration of feasibility rather than a definitive validation of the approach. To ensure that AI-enabled discovery meaningfully advances personalized therapy, future pipelines should adopt a hybrid translational structure that combines computational generation, targeted experimental verification, and distributed clinical learning. This approach aligns model prediction with patient-level variation, providing a practical path toward personalized and equitable therapeutics.

The broader impact of computationally guided drug discovery will depend not solely on accelerated timelines but on the incorporation of rigorous methodological frameworks. Sustained clinical translation will require that algorithmically generated candidates be embedded within hybrid validation pipelines, wherein computational predictions are iteratively refined and empirically corroborated through structured preclinical testing and prospective clinical evaluation. Furthermore, the adoption of standardized metrics for interpretability, reproducibility, and model transparency will be essential to secure the confidence of regulatory authorities, clinical stakeholders, and patients. Equally, the establishment of internationally harmonized regulatory pathways will be necessary to ensure equitable access to such therapeutics across healthcare systems with varying levels of infrastructure and resources. Absent these considerations, the technical velocity of computational design may exceed the evidentiary standards required for safe and generalizable implementation.

If appropriately integrated, however, AI-enabled platforms may evolve from adjunctive tools to integrative coordination frameworks capable of coordinating multi-omics data, real-time clinical feedback, and adaptive therapeutic optimization. Such a paradigm would support the development of more effective and safer therapies within clinically realistic timelines with the potential to meaningfully influence the scope and cadence of translational medicine.

Importantly, the frameworks validated in this early trial may be particularly impactful for oncology. Cancer drug development is uniquely challenged by tumor heterogeneity, clonal evolution, and acquired resistance, creating an urgent need for adaptive and data-driven strategies. As integrative coordination frameworks, AI-enabled platforms can uniquely synthesize multi-omics data and real-time clinical feedback to address these complexities. Such a paradigm will facilitate the delivery of safer, more precise anticancer therapies, ultimately advancing the landscape of translational oncology.

## Data Availability

No new data were generated or analyzed in support of this research.
